# Supplementary material for: Dysfunction of peripheral somatic and autonomic nervous system in patients with severe forms of Crohn’s disease on biological therapy with TNFα inhibitors–A single center study
Source: PLoS One. 2023 Nov 15;18(11):e0294441. doi: 10.1371/journal.pone.0294441 (PMC10650985; doi:10.1371/journal.pone.0294441)
Supplement: S1 File — (DOCX) [file pone.0294441.s001.docx]

**The sensor-motor neuropathy questionnaire**

1. Do you feel a worsening of sensitivity in your hands or feet?

YES NO

(1 point) (2 points)

1. Have you ever felt burning pain in your feet?

YES NO

(1 point) (2 points)

1. Do you feel heavy and weak in your legs?

YES NO

(1 point) (2 points)

1. Are your feet hypersensitive to touch?

YES NO

(1 point) (2 points)

1. Have you ever had stabbing or stinging in your feet?

YES NO

(1 point) (2 points)

1. Do you feel pain even when the blanket touches your skin?

YES NO

(1 point) (2 points)

1. Are you able to identify the localization of the pain?

YES NO

(1 point) (2 points)

1. Are you able to distinguish between hot and cold water when bathing?

YES NO

(1 point) (2 points)

1. Has a doctor ever told you that you have neuropathy?

YES NO

(1 point) (2 points)

1. Are your symptoms worse at night?

YES NO

(1 point) (2 points)

1. Is the skin on your feet so dry that it develops tears?

YES NO

(1 point) (2 points)

1. Did you have an amputation?

YES NO

(1 point) (2 points)
