# Supplementary material for: Dysfunction of peripheral somatic and autonomic nervous system in patients with severe forms of Crohn’s disease on biological therapy with TNFα inhibitors–A single center study
Source: PLoS One. 2023 Nov 15;18(11):e0294441. doi: 10.1371/journal.pone.0294441 (PMC10650985; doi:10.1371/journal.pone.0294441)
Supplement: S2 File — (DOCX) [file pone.0294441.s002.docx]

**The autonomic neuropathy questionnaire**

1. **Cardiovascular system:**
2. Do you have palpitations?
3. Do you faint or feel dizzy after standing?
4. **Gastrointestinal system:**
5. Do you have a feeling of fullness after eating or difficulty swallowing or hiccups?
6. Do you have difficulty defecating - diarrhea or constipation?
7. Do you have constipation or diarrhea?
8. **Urogenital system:**
9. Do you have trouble with incontinence of your urine?
10. Do you have any other problém with urination?
11. Do you have any problem in your sexual life (e.g. erectile dysfunction)?
12. **Skin:**
13. Do you experience sweating after meals or at night?
14. Do you sweat noticeably more on the upper half of your body?
15. Do you not sweat on your legs?
16. Do you find it difficult to tolerate heat and higher temperatures?
