# Supplementary material for: Dysfunction of peripheral somatic and autonomic nervous system in patients with severe forms of Crohn’s disease on biological therapy with TNFα inhibitors–A single center study
Source: PLoS One. 2023 Nov 15;18(11):e0294441. doi: 10.1371/journal.pone.0294441 (PMC10650985; doi:10.1371/journal.pone.0294441)
Supplement: Attachment — Submitted filename: Response to reviewers.docx [file pone.0294441.s004.docx]

Dear reviewers and editors,

First of all, I would like to thank you very much for your beneficial recommendations and interesting comments to our manuscript. All your comments were reflected during revision of our manuscript.

Below I would like to present you these manuscript changes in details:

**Journal Requirements:**

# 1. Please ensure that your manuscript meets PLOS ONE's style requirements, including those for file naming.

- **Manuscript was checked and corrected based on journal requirements according to sent pdf documents.**

# 2. We note that the grant information you provided in the ‘Funding Information’ and ‘Financial Disclosure’ sections do not match. When you resubmit, please ensure that you provide the correct grant numbers for the awards you received for your study in the ‘Funding Information’ section.

- **Grant information will be checked during resubmitting of the revised version of our manuscript.**
- **Based on the internal requirements of our faculty (for reasons of financing the publication process), we would like to ask you to cite our funding statement (Supported by the Ministry of Health, Czech Republic - conceptual development of research organization, Motol University Hospital, Prague, Czech Republic 00064203.) somewhere as part of our manuscript.**

# 3. Please review your reference list to ensure that it is complete and correct. If you have cited papers that have been retracted, please include the rationale for doing so in the manuscript text, or remove these references and replace them with relevant current references. Any changes to the reference list should be mentioned in the rebuttal letter that accompanies your revised manuscript. If you need to cite a retracted article, indicate the article’s retracted status in the References list and also include a citation and full reference for the retraction notice.

# All references were checked (if you have a problem with some specific reference, please notify us of this and we will certainly reflect that).

# Reviewer's Comments to the Author

**Reviewer #1:**

This manuscript investigated neurological issues in severe Crohn's disease patients on anti-TNFα therapy. It found a high prevalence of peripheral neuropathy (36.7%) and cardiovascular autonomic neuropathy (56.7%). Risk factors included age, therapy duration, BMI, and disease activity. The study also highlighted the importance of neurological assessments in these patients. However，in the manuscript, there are areas where refinement and improvement are still necessary.

Comments:

1. The figure legend needs to be supplemented in detail, for example, the numbers on the bars in Figure 1 represent the number of individuals, and it would be best if the statistical differences (p-values) could be reflected on the figure.

- **Figure legends were implemented in details according to your beneficial recommendation.**
- **Figure 1 summarizes the results of the four main types of measurements in our study; correlations were not included in the figure mainly due to the small number of patients in our study. The graph in Figure 1 was modified in response to your comments for better reproducibility of results - data labels of results, vertical axis, percentages of normal and pathological measurement results.**

2. Please present the results of the two questionnaires in the form of tables or figures

- **Manuscript was edited based on your requirements, the table with the results of both questionnaires was added to the manuscript.**

3. Many of the data descriptions in the manuscript cannot be directly obtained from the provided tables and figures. Please provide additional information, for example, but not limited to the results of blood samples and the data from lines 296 to 302.

- **Manuscript was edited based on your requirements, table with source data of our study was added as the separate additional file with our resubmitted manuscript.**

**Reviewer #2:**

The submitted manuscript has done an excellent job of demonstrating a high prevalence of peripheral neuropathy in patients with severe Crohn's disease on anti-TNF alpha therapy. Specific risk factors have also been presented which are responsible for the development of this neuropathy in patients. Overall the manuscript is very detailed, well explained and the data has been presented in a clear manner. However, there are certain minor errors which need to be addressed:

1. Page 4, Line 58 : The spelling of "inflamatory" is incorrect.

- **Manuscript was corrected according to your recommendation.**

2. Page 6, Line 101 : It is suggested to replace the word "realized" with "conducted".

- **Manuscript was corrected according to your recommendation.**

3. Page 6, Line 107 : Please keep the format of anti-TNF alpha consistent.

- **Manuscript was corrected according to your recommendation.**

4. Page 7, Line 131 : What are "ionts"?

- **Ionts were incorrectly mentioned in the text instead of electrolytes, manuscript was corrected.**

5. Page 7, Line 135 : "Coeliac Disease" is misspelled.

- **Manuscript was corrected according to your recommendation.**

6. Page 14, Line 276 : The spelling of "melittus" is incorrect.

- **Manuscript was corrected according to your recommendation.**
